# *Mycoplasma pneumoniae* detections in children with acute respiratory infection, 2010–2023: a large sample study in China

**DOI:** 10.1186/s13052-025-01846-7

**Published:** 2025-01-23

**Authors:** Yuzhu Miao, Jungen Li, Linlin Huang, Ting Shi, Tingbo Jiang

**Affiliations:** 1 Department of Cardiovascular Medicine, The First Affiliated Hospital of Soochow University, Suzhou, 215000 China; 2 Department of Emergency Medicine, The First Affiliated Hospital of Soochow University, Suzhou, China; 3 Pediatric Intensive Care Unit, Children’s Hospital of Soochow University, Suzhou, China; 4 Department of Infectious Diseases, Children’s Hospital of Soochow University, Suzhou, China

**Keywords:** *Mycoplasma pneumoniae*, Children, Epidemiology, NPIs

## Abstract

**Background:**

This study aimed to describe the epidemiological trends of *Mycoplasma pneumoniae* (MP) infection among children with acute respiratory tract infections (ARTIs) before, during and after the COVID-19 pandemic, and evaluating the impact of non-pharmaceutical interventions (NPIs) on the epidemiology of MP infection.

**Methods:**

Children with ARTIs admitted to the Children’s Hospital of Soochow University (SCH) from January 2010 to December 2023 and underwent MP nucleic acid PCR assay were included. Clinical data on age, sex, onset time and detection result were collected and analyzed.

**Results:**

All of the 122,984 inpatients were enrolled, in which 20.8% (25659/122984) of the children with MP tested positive, including 19.4% (14139/72662) for male and 22.9% (11520/50322) for female. It was a statistically significant difference between the two genders (*p* < 0.05). In addition, the positive rate of MP was the highest in the age group > 6 years old each year (*p* < 0.05). During 14-year period, the detection rate of MP has experienced four peaks in 2012, 2013, 2019, and 2023. Before the NPIs the prevalence of MP showed seasonality, and the number and rate of MP positivity reached their peak in August. However, the rate of MP positivity remained at a low level during the NPIs. After the abolition of NPIs, the MP positivity rate obviously increased and remained at a high level.

**Conclusions:**

The NPIs could reduce the spread of MP infection and change its epidemic season, but it has not changed the susceptible population of MP infection.

**Supplementary Information:**

The online version contains supplementary material available at 10.1186/s13052-025-01846-7.

## Introduction

As the smallest prokaryotic cell-type microorganism, *Mycoplasma pneumoniae* (MP) has no cell wall and is located between bacteria and viruses. It is an important etiological factor of upper and lower respiratory infections in children [1]. In recent years, MP has generally replaced Streptococcus pneumoniae as the most common bacterial pathogen causing respiratory diseases in children [2]. MP may account for 30–50% of community-acquired pneumonia (CAP) cases with increasing during epidemic, of which 18% require hospitalization [3]. In addition, it was estimated that 2 million MP infection cases occurred each year in the United States [4]. The MP epidemic has brought a heavy economic burden to families and society, while posing a huge challenge to medical resources.

Since 2020, the government took strict non-pharmaceutical interventions (NPIs) such as the prohibition of social gatherings and wearing masks to cut off the spread of coronavirus disease 2019 (COVID-19). Although NPIs mainly focused on the prevention of COVID-19 cases, it also reduced other respiratory pathogens infection, especially in children [5]. Before the COVID-19 pandemic, worldwide incidence of MP was 8.61% between 2017 and 2020, which was measured by direct assay methods. However, during NPIs period the incidence decreased to 1.69% from 2020 to 2021 [6]. After 2022, several countries gradually cancelled the NPIs measures, MP infection showed a delayed outbreak trend in Northern China and Arkansas [7, 8]. Most studies [9, 10]attributed the resurgence of MP infection in children to immune liability, but is it really just because of this? Or is it related to the epidemic pattern of the MP itself?

In recent years, the prevalence of MP infection in children in Suzhou, China has not been explored. This study aimed to describe the epidemic trend of Mycoplasma infection in children with acute respiratory tract infections (ARTIs) from January 2010 to December 2023 based on nucleic acid testing, which was meaningful for an overall knowledge of MP prevalence among children. Monitoring the changes can not only provide a reference for public health decision-making but also help prepare for the potential outbreak of Mycoplasma.

## Materials and methods

### Participants

This retrospective study investigated the detection rate of *Mycoplasma pneumoniae*(MP) in children admitted to the Children’s Hospital of Soochow University (SCH), the only tertiary hospital located in Suzhou. Based on the hospital audit report (2010–2023), we received an average of 63,000 inpatients per year. This study spanned fourteen years, from 1st January 2010 to 31st December 2023. The hospitalized patients ranging from 1 month to 16 years old who presented with acute respiratory tract infections (ARTIs) and underwent MP nucleic acid PCR assay were included(flowchart see Supplementary Material, Figure S1). The information of participants’ sex, age, onset time and results for MP nucleic acid PCR were collected using the electronic medical system.

### Specimen collection

Nasopharyngeal aspirates were obtained from hospitalized patients with suspected ARTIs. The specimens were collected into suctioning tubes and stored at 2–8℃ for examination within 30 min.

### Real-time fluorescent quantitation PCR for MP

The aspirate was shaken for 30 s, centrifuged at 15,000 g for 5 minutes, the supernatant was removed, and the lysis solution was added to extract DNA. The DNA-PCR amplification was then performed on the iCycler iQ fluorescence quantitative PCR instrument(Bio-Rad, California, USA) under the following conditions: 37℃ for 2 min, 94℃ for 10 min, and 40 cycles of 94℃ for 10s, 55℃ for 30 s, and 72℃ for 40 s. The primers and probes were obtained from Guangzhou Da’an Gene Co. For each assay, a negative, critical, positive control, and four positive quantity controls (10^5^, 10^6^, 10^7^, and 10^8^ copies/mL) were used. Follow the manufacturer’s instructions for all operations.

### Definition of ARTIs

The definition of ARTIs includes acute upper respiratory infections and acute lower respiratory infections, which can be caused by viruses, bacteria, atypical pathogens, etc [11].

### Statistical analysis

The categorical variables were expressed as percentages and continuous data as mean. Categorical variables were compared using the chi-squared test. The MP data was divided into three cohorts: pre- NPIs (2010–2019), during- NPIs (2020–2022) and post- abolition of NPIs (2023). To visualize the trends each cohort, the data of 2010–2019 presented as mean and 95% confidence interval(CI) and data of 2020–2022 presented as median (minimum to maximum). All statistical analyses were mapped using GraphPad Prism version 9 and conducted using IBM SPSS Statistics. Statistical significance between variables were set at *p* < 0.05.

## Results

### Characteristics of the participant children with ARTIs

All of the 122,984 patients underwent MP-DNA PCR detection, including 72,662 males (59.1%) and 50,322 females (40.9%). In this study, 55,291 (45.0%) children were under 1 year old, 28,934(23.5%) were 1–3 years old, 22,522(18.3%) were 3–6 years old and 16,237 (13.2%) were over 6 years old. As shown in Table 1, the age distributions of the participants similar in 2010–2022, with the majority of cases ≤ 3 years old (especially ≤ 1 years). However, in 2023, the most common cases were among children aged>6 years (*P*<0.05).

**Table 1:** Demographics of the enrolled patients with ARTIs

Category	2010 (*n* = 5046)	2011 (*n* = 6127)	2012 (*n* = 8489)	2013 (*n* = 8504)	2014 (*n* = 7753)	2015 (*n* = 6963)	2016 (*n* = 6393)	2017 (*n* = 7722)	2018 (*n* = 11262)	2019 (*n* = 16979)	2020 (*n* = 6623)	2021 (*n* = 9210)	2022 (*n* = 7515)	2023 (*n* = 14398)
Sex (male, n%)	3201 (63.4)	3939 (64.2)	5293 (62.3)	5272 (62.0)	4794 (61.8)	4148 (59.5)	3801 (59.4)	4639 (60.1)	6713 (57.7)	9628 (56.7)	3897 (58.8)	5315 (57.7)	4291 (57.1)	7731 (53.7)
Age (years, n%)														
≤ 1	2798 (55.4)	4238 (69.2)	4997 (58.8)	4804 (56.5)	4504 (58.1)	3910 (56.2)	3126 (48.9)	4114 (53.3)	5418 (48.1)	6268 (36.9)	2885 (43.5)	3483 (37.8)	2281 (30.4)	2465 (17.1)
1-≤3	1092 (21.6)	990 (16.1)	1763 (20.8)	1790 (21.0)	1711 (22.0)	1544 (22.2)	1534 (24.0)	1654 (21.4)	2887 (25.6)	4565 (26.9)	1838 (27.8)	2897 (31.5)	2183 (29.0)	2486 (17.3)
3-≤6	754 (15.0)	588 (9.6)	1054 (12.4)	1153 (13.6)	942 (12.2)	1003 (14.4)	1185 (18.5)	1302 (16.9)	2031 (18.0)	3701 (21.8)	1179 (17.8)	1937 (21.0)	1893 (25.2)	3800 (26.4)
>6	402 (8.0)	311 (5.1)	675 (8.0)	757 (8.9)	596 (7.7)	506 (7.2)	548 (8.6)	652 (8.4)	926 (8.2)	2445 (14.4)	721 (10.9)	893 (9.7)	1158 (15.4)	5647 (39.2)
*P*	<0.05	<0.05	<0.05	<0.05	<0.05	<0.05	<0.05	<0.05	<0.05	<0.05	<0.05	<0.05	<0.05	<0.05

*Notes* The chi-square test for categorical variables

### Positive rate of MP in children with ARTIs each year

**Fig. 1:**
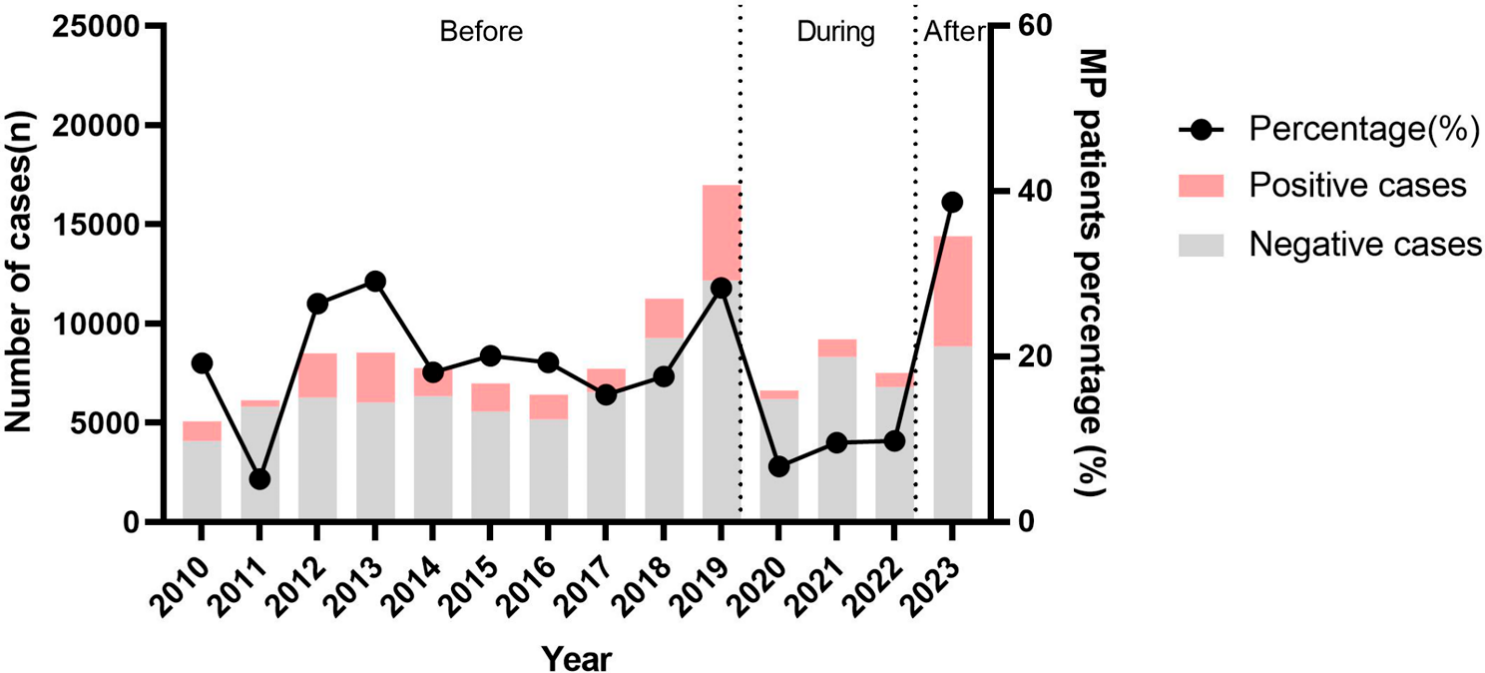
The number of specimens tested and positive rate of MP between January 2010 and December 2023

In total, 20.8% of the children with ARTIs tested positive for MP, the percentage of males and females was 19.4% (14139/72662) and 22.9% (11520/50322). It was a statistically significant difference between the two genders (*p* < 0.05). In addition, Table 2 showed that positive rate of MP was the highest in the age group > 6 years old each year (*p* < 0.05). As shown in Fig. 1, the number of MP test cases reached its peak in 2019 before NPIs, then it was at a low level during NPIs. After the policy was cancelled, the number of test cases gradually increased and remained at a high level. The positive rates of MP from 2010 to 2023 were 19.2% (970/5046), 5.2% (316/6127), 26.4% (2239/8489), 29.1% (2472/8504), 18.1% (1406/7753), 20.1% (1397/6963), 19.3% (1237/6393), 15.4% (1191/7722), 17.6% (1984/11262), 28.3% (4808/16979), 6.7% (442/6623), 9.6% (888/9210), 9.8% (734/7515) and 38.7% (5575/14398), respectively. Moreover, the detection rate of MP has experienced four peaks in the past 14 years, namely in 2012, 2013, 2019, and 2023.

**Table 2:** Demographics of the inpatients with MP infection

Category	2010 (*n* = 970)	2011 (*n* = 316)	2012 (*n* = 2239)	2013 (*n* = 2472)	2014 (*n* = 1406)	2015 (*n* = 1397)	2016 (*n* = 1237)	2017 (*n* = 1191)	2018 (*n* = 1984)	2019 (*n* = 4808)	2020 (*n* = 442)	2021 (*n* = 888)	2022 (*n* = 734)	2023 (*n* = 5575)
Sex (n%)														
Male	547 (17.1)	197 (5.0)	1358 (25.7)	1435 (27.2)	815 (17.0)	774 (18.7)	684 (18.0)	686 (14.8)	1110 (16.5)	2562 (26.6)	245 (6.3)	477 (9.0)	397 (9.3)	2852 (36.9)
Female	423 (22.9)	119 (5.4)	881 (27.6)	1037 (32.1)	591 (20.0)	623 (22.1)	553 (21.3)	505 (16.4)	874 (19.2)	2246 (30.6)	197 (7.2)	411 (10.6)	337 (10.5)	2723 (40.8)
*P*	<0.05	0.458	0.053	<0.05	<0.05	<0.05	<0.05	0.058	<0.05	<0.05	0.132	<0.05	0.083	<0.05
Age (years, n %)														
≤ 1	255 (9.1)	84 (2.0)	854 (17.1)	832 (17.3)	452 (10.0)	466 (11.9)	340 (10.9)	351 (8.5)	496 (9.2)	785 (12.5)	88 (3.1)	91 (2.6)	57 (2.5)	243 (9.9)
1-≤3	215 (19.7)	70 (7.1)	517 (29.3)	574 (32.1)	304 (17.8)	315 (20.4)	274 (17.9)	237 (14.3)	431 (14.9)	1102 (24.1)	73 (4.0)	194 (6.7)	127 (5.8)	534 (21.5)
3-≤6	289 (38.3)	96 (16.3)	447 (42.4)	568 (49.3)	331 (35.1)	340 (33.9)	370 (31.2)	319 (24.5)	596 (29.3)	1512 (40.9)	106 (9.0)	270 (13.9)	217 (11.5)	1409 (37.1)
>6	211 (52.5)	66 (21.2)	421 (62.4)	498 (65.8)	319 (53.5)	276 (54.5)	253 (46.2)	284 (43.6)	461 (49.8)	1409 (57.6)	175 (24.3)	333 (37.3)	333 (28.8)	3389 (60.0)
*P for trend*	<0.05	<0.05	<0.05	<0.05	<0.05	<0.05	<0.05	<0.05	<0.05	<0.05	<0.05	<0.05	<0.05	<0.05

*Notes* Percentage (%) = number of positive MP /total number with test. For example, the percentage of male with MP = number of males with MP /total number of males with MP test. The chi-square test for categorical variables, and t- test for continuous variables

### Monthly distributions of MP

**Fig. 2:**
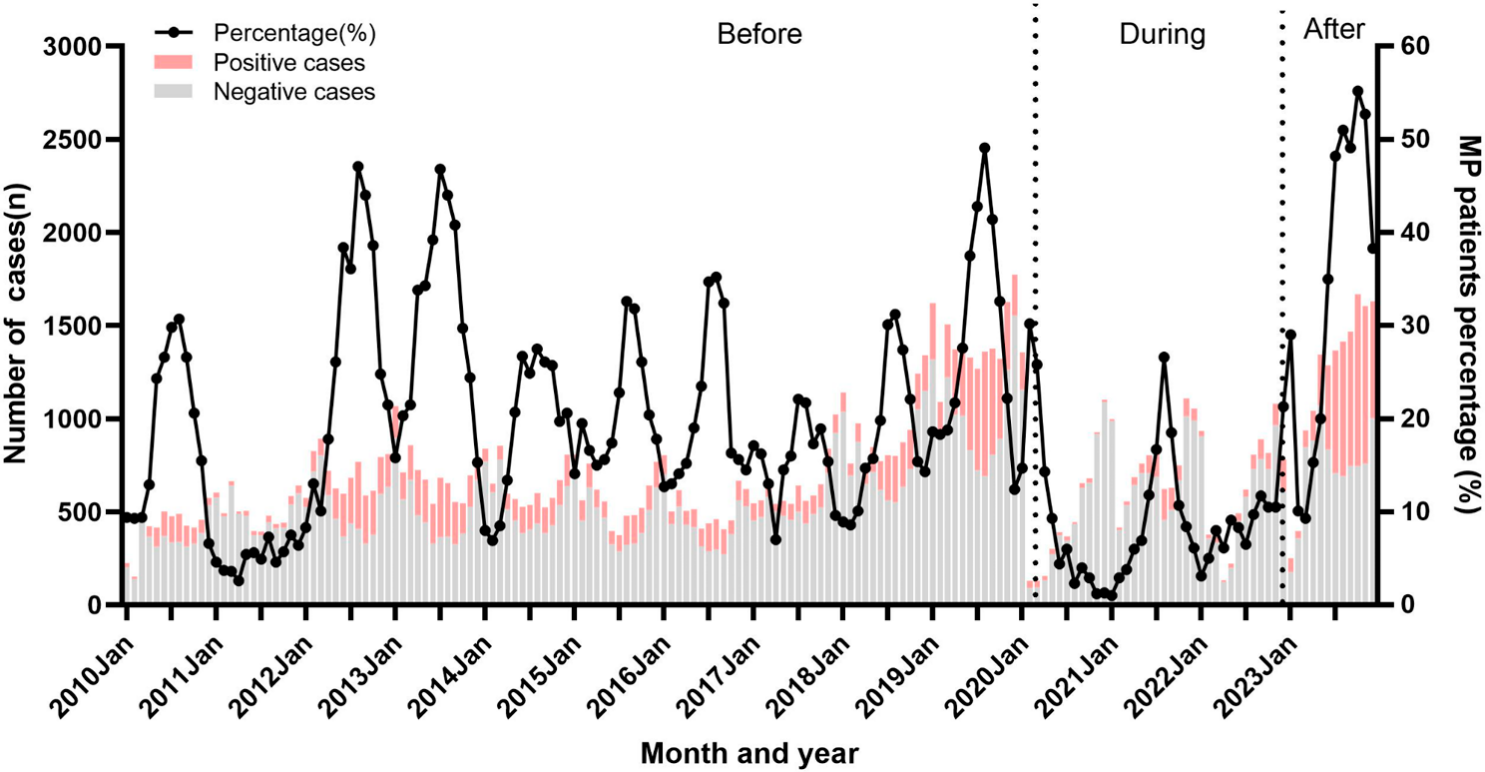
The monthly number and positive rate of MP from January 2010 to December 2023

**Fig. 3:**
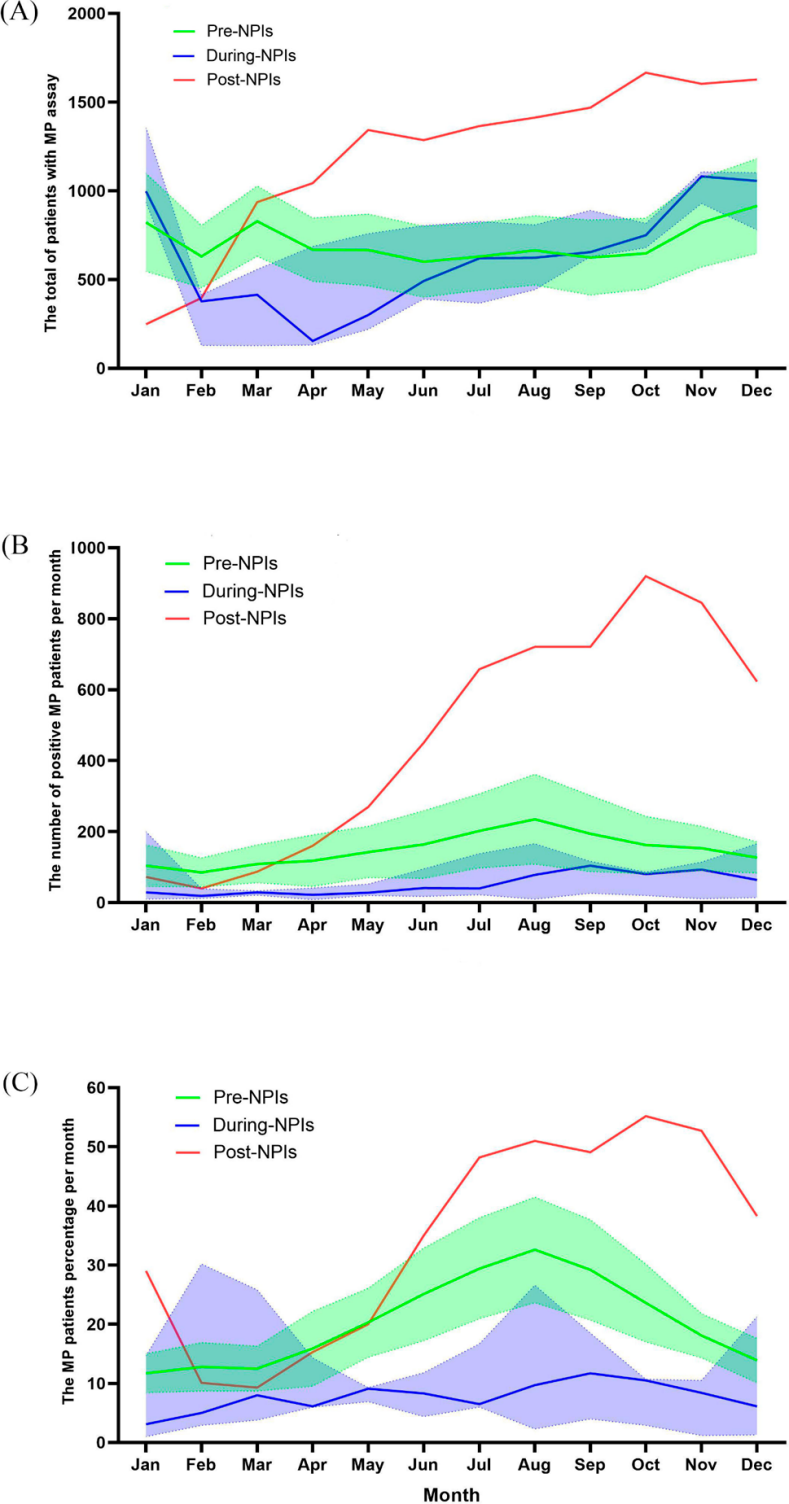
Compare the monthy number and positive rate of MP detection pre-NPIs, during-NPIs and post-NPIs. Green line: mean 2010–2019, green shade: 95% confidence interval 2010–2019, purple line: median 2020–2022, green shade: minimum to maximum, red line: absolute number per month from 2023. **(A)** Total number of patients per month with MP DNA-PCR assay; **(B)** Total number of positive MP assay per month; **(C)** Positive rate of MP per month with DNA PCR testing for MP

Before the NPIs, the prevalence of MP showed seasonality and the peak positive rate of MP in all years was from July to September, except for 2011 (Fig. 2). During the NPIs, the positive rate of MP remained at a low level, except for a small peak in 2021. After the policy was cancelled, the prevalence of MP called off its seasonality, and the positivity rate of MP began to rise in early January 2023, reaching its peak in October, and has since remained at a high level. As shown in Fig. 3, the prevalence of MP showed seasonality and the number and rate of MP positivity reached their peak in August before the NPIs. During the NPIs, the number and rate of MP positivity remained at a low level. After the abolition of NPIs, the number of MP test and positivity, and MP positivity rate obviously increased, and the detection peak of MP was apparently prolonged.

## Discussion

This study investigated the epidemiological trends of MP infection before, during and after COVID-19 pandemic. In addition, the analysis of 14 years clinical data showed that the number of children with acute respiratory tract infections (ARTIs) who underwent MP DNA-PCR assay sharply rase in 2019 and 2023. However, it plummeted sharply and remained at a low level during COVID-19 pandemic (from 2020 to 2022), which indicated that non-pharmaceutical interventions (NPIs) have to some extent reduced the spread of acute respiratory diseases, which consisted with previous studies [5, 11]. Interestingly, the inpatients with ARTIs were more commonly infants from 2010 to 2022. However, following the discontinuation of strict NPIs, the trend shifted towards school-aged children (6–16years). The reason may be that young children had poor compliance with NPIs, while older children were more socially active after the discontinuation of NPIs [12].

In this study, the proportion of ARTIs caused by MP varies by age, with school-age (>6 years) children being the most common age group affected during the 14 years (before, during and after COVID-19 pandemic), which was consist with previous studies [1, 8]. In addition, girls were more susceptible to MP than boys in this study, which was also consistent with the previous research by Cheng et al. and Zhang et al. [13, 14]. The reason was believed to be related to the different lifestyles of them that boys spend more time engaging in outdoor activities than girls [13]. Overall, the NPIs have not changed the demographic characteristics of MP infection in children.

Previous studies indicated that MP infection complied with an epidemic cycle of approximately 3–7 years, with each outbreak spanning 1–2 years and can occur throughout the year [4]. In this study, there were epidemic of MP in 2012 and 2013. According to the trend of MP, the recent MP epidemic was expected to prolong from 2019 to 2021 [15, 16]. In fact, there was indeed a pandemic of MP in Suzhou, China in 2019. However, compared to 2019, the positive cases and detection rates of MP in 2020, 2021 and 2022 have marked declined. It suggested that strict NPIs have the potential to remarkable reduce the prevalence of MP in semi-closed or closed populations [17, 18]. Since cancellation of NPIs in December 2022 in Suzhou, China, the detection rate of MP was at a low level from January to February 2023, but it continued to rise until June 2023 and persisted until the end of the investigation. The slow generation time (6 h) and transmission (1–3 weeks incubation period) of MP result in a longer time interval than the virus to re-establishment in the population after stopping NPIs [19]. So, the cancellation of NPIs did not immediately lead to the spread of MP infection in the population. However, due to the low MP infection rate for 3 years, the population lacked immune protection (also known as immune debt) [9], and was susceptible to infection, leading to a gradual increase in the number of MP infections in 2023.

According to the monthly MP-positivity rates, a peak was in August of each year before the COVID-19 epidemic, except 2011. It indicated that the prevalence of MP showed seasonal variation. Previous studies have shown that the peak of MP detection in eastern China (such as Zhejiang) was in summer and autumn [9], while in northern China was in winter [20]. A research report from Japan stated that there was a positive correlation between rising temperatures and the occurrence of MP infection, which also explains why the number of infections increases during warmer months [21, 22]. However, the diversity of MP epidemic seasons appearing in the north and south of China may be related to the living environment, public health measures, and activity patterns [4]. It is worth noting that the detection rate of MP infection in 2011 in this study remained low throughout the year without a peak. Considering the local epidemiological characteristics, it may be due to the high incidence of hand, foot, and mouth disease [23] and the competition between pathogens.

When implementing NPIs and lifting the policy within one year, the prevalence of MP infection showed no obvious seasonal, which was consistent with previous research [24, 25]. It indicates that NPIs can not only reduce the MP infection in children, but also interfere with its seasonal characteristics.

## Conclusions

we compared the data pre-NPIs, during-NPIs and post-NPIs for fourteen years. We found that MP infection followed an epidemic cycle of approximately 5–7 years, with outbreaks lasting for 2 years and detection peaks occurring in August each year in Suzhou, China. The NPIs could reduce the spread of MP infection and change its epidemic season, but it has not changed the susceptible population of MP infection. In addition, when NPIs were canceled, there was another delayed outbreak due to immune debt.

## Electronic supplementary material

Below is the link to the electronic supplementary material.

Supplementary Material 1

## Data Availability

The datasets generated during and/or analyzed during the current study are available from the corresponding author on reasonable request.

## Abbreviations

MP: *Mycoplasma pneumoniae*
ARTIs: Acute respiratory tract infections
SCH: Children’s Hospital of Soochow University
CAP: Community-acquired pneumonia
NPIs: Non-pharmaceutical interventions
CI: Confidence interval
