# Supplementary figures and images for: Mycoplasma pneumoniae detections in children with acute respiratory infection, 2010–2023: a large sample study in China
Source: Ital J Pediatr. 2025 Jan 23;51:11. doi: 10.1186/s13052-025-01846-7 (PMC11755796; doi:10.1186/s13052-025-01846-7)

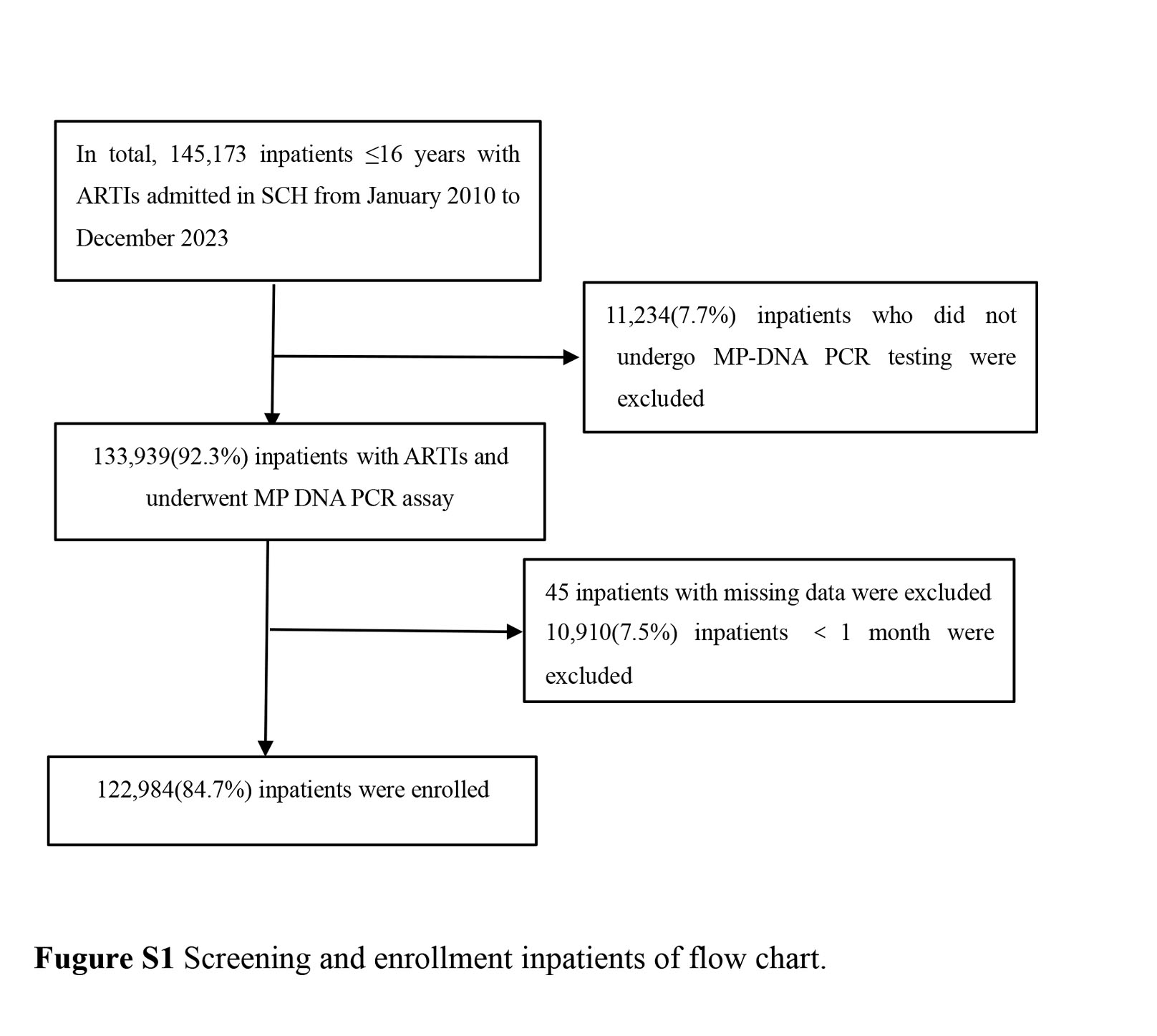

Supplement: Supplementary file 1 — Supplementary Material 1 [file 13052_2025_1846_MOESM1_ESM.jpg]
